# Can a Top Predator Persist After One of the World's Largest Mining Dam Failures? Occupancy Dynamics of the Neotropical Otter in Brazil's Paraopeba River

**DOI:** 10.1002/ece3.73366

**Published:** 2026-04-07

**Authors:** Rodrigo Lima Massara, Paloma Marques Santos, Rodolfo Stumpp, Aline Saturnino Costa, Joyce Ramos Rodrigues, Ana Yoko Ykeuti Meiga, Ricardo R. C. Solar, Mariana Neves Moura, Tiago Dornas, Cristiane Cäsar, Adriano Paglia

**Affiliations:** ^1^ Laboratório de Ecologia e Conservação, Departamento de Genética, Ecologia e Evolução, Universidade Federal de Minas Gerais Belo Horizonte Brazil; ^2^ Instituto de Pesquisa e Conservação de Tamanduás no Brasil Campo Grande Brazil; ^3^ Amplo Engenharia e Gestão de Projetos Ltda Belo Horizonte Brazil; ^4^ School of Natural Resources and Environment University of Florida Gainesville Florida USA; ^5^ Departamento de Genética, Ecologia e Evolução Universidade Federal de Minas Gerais Belo Horizonte Brazil; ^6^ Vale S.A., Diretoria de Reparação Nova Lima Brazil

**Keywords:** ecological persistence, freshwater mammals, habitat use, multi‐season occupancy, tailings impact

## Abstract

Human activities are known to directly impact vertebrate populations worldwide. Among these disturbances, mining dam collapses pose serious threats to freshwater ecosystems, yet their ecological effects have only started to be understood, not only for semi‐aquatic mammals but for biodiversity and ecosystem functioning overall. We assessed post‐disturbance occupancy dynamics of the neotropical otter ( *Lontra longicaudis* ) in the Paraopeba river, Brazil. Over five monitoring seasons (2021–2025), we surveyed five river stretches upstream and downstream of the Ferro‐Carvão confluence and fitted multi‐season occupancy models. We estimated initial occupancy, colonization, extinction, and detection probabilities as functions of distance to the confluence, time since the collapse, water flow velocity, turbidity, and the proportion of natural riparian cover. Our study reveals that otters maintained a continuous distribution and high occupancy probabilities across the monitored river, including stretches affected by tailings. Occupancy dynamics were primarily associated with distance from the confluence, while other environmental variables showed no effects, consistent with a degree of species' tolerance to moderate habitat alterations. Otter occupancy was consistently lowest within the hydroelectric reservoir stretch, suggesting distinct constraints in impounded environments. Our findings demonstrate the post‐disturbance persistence and tolerance of *L. longicaudis* to moderate habitat alteration following a large‐scale environmental disaster and provide important guidance for conservation and management of freshwater systems affected by mining and other anthropogenic disturbances.

## Graphical abstract

We evaluated the effects of the 2019 Brumadinho tailings‐dam collapse on neotropical otters ( *Lontra longicaudis* ) in Brazil's Paraopeba river using multi‐season occupancy models. Six years post‐collapse, otters showed high occupancy across impacted reaches. Lower occupancy in the hydroelectric reservoir underscores species persistence but habitat‐specific limitations.

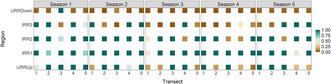

## 1 Introduction

The global biodiversity crisis poses a severe threat to life on Earth, with nearly one million species currently threatened with extinction (IPBES 2019). An extensive review of more than 32,000 populations of over 5200 vertebrate species since 1970 revealed an average population decline of ~70%, particularly severe in tropical areas (WWF 2022). It is estimated that the historical territories of around 8850 out of about 30,000 vertebrate species have undergone substantial range contractions (Ceballos et al. 2017). For mammals specifically, population declines are primarily driven by human‐mediated factors, such as habitat destruction and fragmentation, poaching, environmental degradation, and pollution (Brodie et al. 2021). Beyond these chronic factors, sudden environmental disasters, such as mining dam failures or oil spills, constitute acute and unpredictable disturbances, underscoring the urgent need to understand their ecological impacts more thoroughly.

For example, mining dam collapses are among the most severe anthropogenic disturbances affecting freshwater ecosystems, often resulting in the sudden release of large volumes of tailings that alter river morphology, water quality, and habitat structure (Fernandes et al. 2016; Costa et al. 2022; Pereira et al. 2024). Such events can increase turbidity, modify flow regimes, smother benthic habitats, and disrupt aquatic food webs, with cascading effects on fish communities and higher trophic levels (Fernandes et al. 2016; Costa et al. 2022; Pereira et al. 2024). Wildlife associated with riverine environments may experience both immediate and long‐term impacts, including habitat loss, reduced prey availability, and changes in spatial use patterns.

The neotropical otter ( *Lontra longicaudis* , Olfers 1818) is a semi‐aquatic predator distributed throughout Central and South America (Rheingantz et al. 2014). Due to its reliance on freshwater ecosystems and surrounding terrestrial areas, extensive home range, and specialized diet, *L. longicaudis* is a suitable indicator species for anthropogenic environmental changes (Quadros and Monteiro‐Filho 2002). Studies show that the diet of *L. longicaudis* is dominated by fish, with crustaceans as the second most important prey item (Pardini 1998; Rheingantz, de Menezes, et al. 2017). *L. longicaudis* exhibits flexibility in its activity period, being active during both day and night depending on prey availability and human disturbance (Rheingantz, Santiago‐Plata, and Trinca 2017).

The species occupies a wide variety of aquatic habitats, including both lentic (still) and lotic (flowing) systems, and shows some degree of resilience to anthropogenic disturbances (Lanna et al. 2022). *Lontra longicaudis* has been recorded in impacted environments, such as rivers polluted by effluents, regions impacted by agriculture and livestock activities (Coletti et al. 2013; Krug et al. 2019; Rodríguez‐Aguilar et al. 2022), and reservoirs associated with hydroelectric dams (Santos and Reis 2012; Pérez et al. 2020). However, evidence suggests that when anthropogenic stressors exceed the species' tolerance thresholds, negative effects may occur on its habitat use, behavior, and diet (Coletti et al. 2013), including avoidance of areas with intense boat traffic (Gomez et al. 2014) and rivers altered by damming (Quadros 2012). The interaction between neotropical otters and fisheries remains ambiguous, as otters may either be attracted to fishing zones due to increased prey availability (Andrade et al. 2019) or avoid them to reduce human‐related risks (Barbieri et al. 2012). Additional pressures, such as the accumulation of heavy metals in aquatic food webs (Josef et al. 2008; Ramos‐Rosas et al. 2013), may further compromise the species' long‐term persistence. In Brazil, the species is not nationally classified as threatened (MMA 2022), but it is listed as “Vulnerable” in the state of Minas Gerais (COPAM 2010), and since 2020 has been assumed by the IUCN as “Near Threatened,” primarily due to deforestation, water pollution, and alteration of aquatic environments (IUCN 2025). Given these vulnerabilities, assessing the consequences of large‐scale environmental disturbances, such as mining dam collapses, is crucial both for conservation of *L. longicaudis* and for understanding resilience and recovery processes in impacted freshwater ecosystems.

In January 2019, the collapse of the B1 tailings dam at Vale S.A.'s Córrego do Feijão mine in Brumadinho, state of Minas Gerais, released a massive volume of mining waste into the Ferro‐Carvão stream. On the same day, the tailings (~16% of the total volume) reached the Paraopeba river, triggering a sudden influx of mining waste that compounded long‐term land‐use changes, hydrological alterations, and habitat restructuring across the basin (Silva Rotta et al. 2020; Thompson et al. 2020; Laureano et al. 2022). Studies conducted in the weeks and months following the B1 dam collapse documented extreme physicochemical disturbances in the Paraopeba river, including exceptionally high turbidity and elevated concentrations of trace elements in water and sediments, frequently exceeding regulatory thresholds (Vergilio et al. 2020). These alterations were associated with acute ecotoxicological effects across multiple trophic levels, such as impaired algal growth, reduced mobility in microcrustaceans, and metal accumulation in fish tissues, indicating a strong potential for contaminant transfer through the aquatic food web. Importantly, evidence suggests that although the most severe physicochemical impacts occurred shortly after the collapse, contaminant bioavailability persisted beyond the acute phase. Elevated concentrations of bioavailable metals in fish and macrophytes were detected during the first year after the disaster, even where sediment concentrations had declined, indicating prolonged exposure of aquatic biota (Parente et al. 2021). Model‐based projections further suggest that recovery of key physicochemical conditions in the Paraopeba river may require approximately 7–11 years, reflecting the long‐term redistribution and attenuation of mining‐derived contaminants in large river systems (Pacheco et al. 2022).

Together, this body of evidence indicates that the dam failure may affect trophic pathways and prey availability relevant to top predators, such as the neotropical otter. Nevertheless, recent surveys have documented that *L. longicaudis* remains present both upstream and downstream of the collapse site, suggesting persistence of habitat use in the basin during the post‐disturbance period (Lanna et al. 2022).

Because otter monitoring started 2 years after the dam collapse, this study focuses on post‐disturbance dynamics. Accordingly, we evaluated habitat use of neotropical otters along the Paraopeba river following the dam collapse, measured through occupancy probability. Due to the solitary habits of *L. longicaudis* and the challenges associated with direct observation, research often relies on indirect indicators, such as tracks and excrements (Rheingantz, Santiago‐Plata, and Trinca 2017). Occupancy models that account for imperfect detection and false absences are effective tools for evaluating habitat use factors (MacKenzie et al. 2002; Navarro‐Picado et al. 2017; Pocasangre‐Orellana and Parallada 2018; Smith et al. 2020).

Specifically, we analyzed occupancy patterns across local spatial scales (sites at varying distances from the Ferro‐Carvão confluence) and temporal scales (years following the collapse). Regarding spatial scale, we hypothesized that occupancy probability could vary along this longitudinal gradient, reflecting differences in exposure to tailings. On the temporal scale, we expected occupancy to increase over time as restoration efforts and natural ecological recovery mitigated disaster impacts. Conversely, if the collapse had no lasting negative effects, we predicted weak or minimal spatial–temporal variation in occupancy. Additionally, we considered environmental factors unrelated to the dam collapse, such as natural vegetation, water turbidity, and flow velocity that may impact otter occupancy. We expected higher occupancy probability in better preserved river stretches, where natural vegetation remains less disturbed, since *L. longicaudis* , despite tolerating some disturbance (Smith et al. 2020), tends to avoid heavily modified banks (Gomez et al. 2014). Additionally, we anticipated clearer water in sites to favor neotropical otter presence, as turbidity can limit prey detection (Cruz García et al. 2017). The effect of flow velocity remains uncertain, with some studies linking the species to faster‐flowing rivers (Larivière 1999; de Almeida and Pereira 2017), while others suggest a preference for slower flows that enhance foraging efficiency (Cho et al. 2009; Cruz García et al. 2017).

Finally, considering the variables mentioned, we hypothesized that detection probability would increase over time following the disaster and with greater distance from the confluence of the Ferro‐Carvão stream and the Paraopeba river as detection conditions improved.

## 2 Methodology

### 2.1 Study Area and River Reaches

The Paraopeba river is a major tributary of the São Francisco river, located in the central region of the state of Minas Gerais, Brazil. The river basin spans an area of over 12,000 km^2^, representing approximately 5% of the larger São Francisco basin. The main channel of the Paraopeba river extends for approximately 510 km, originating in the municipality of Cristiano Otoni and discharging into the Três Marias Hydroelectric Power Plant reservoir in Felixlândia (CBHSF 2025).

The Paraopeba basin holds strategic importance for the Belo Horizonte metropolitan region, providing a water supply to an estimated 60% of its 3.5 million inhabitants (ANA 2025). However, the river's upper and middle courses are subject to considerable anthropogenic pressure, primarily from intensive iron and manganese mining activities (IGAM 2022). Urban and industrial impacts, particularly from the cities of Contagem and Betim, combined with domestic sewage from smaller towns, further degrade the river's water quality (Soares et al. 2022). Historically, the basin's native Atlantic Forest cover has been reduced from nearly 40% to just 25% due to land use conversion for pastureland (53.5%) and cropland (12.3%) (Mapbiomas 2022).

**FIGURE 1:**
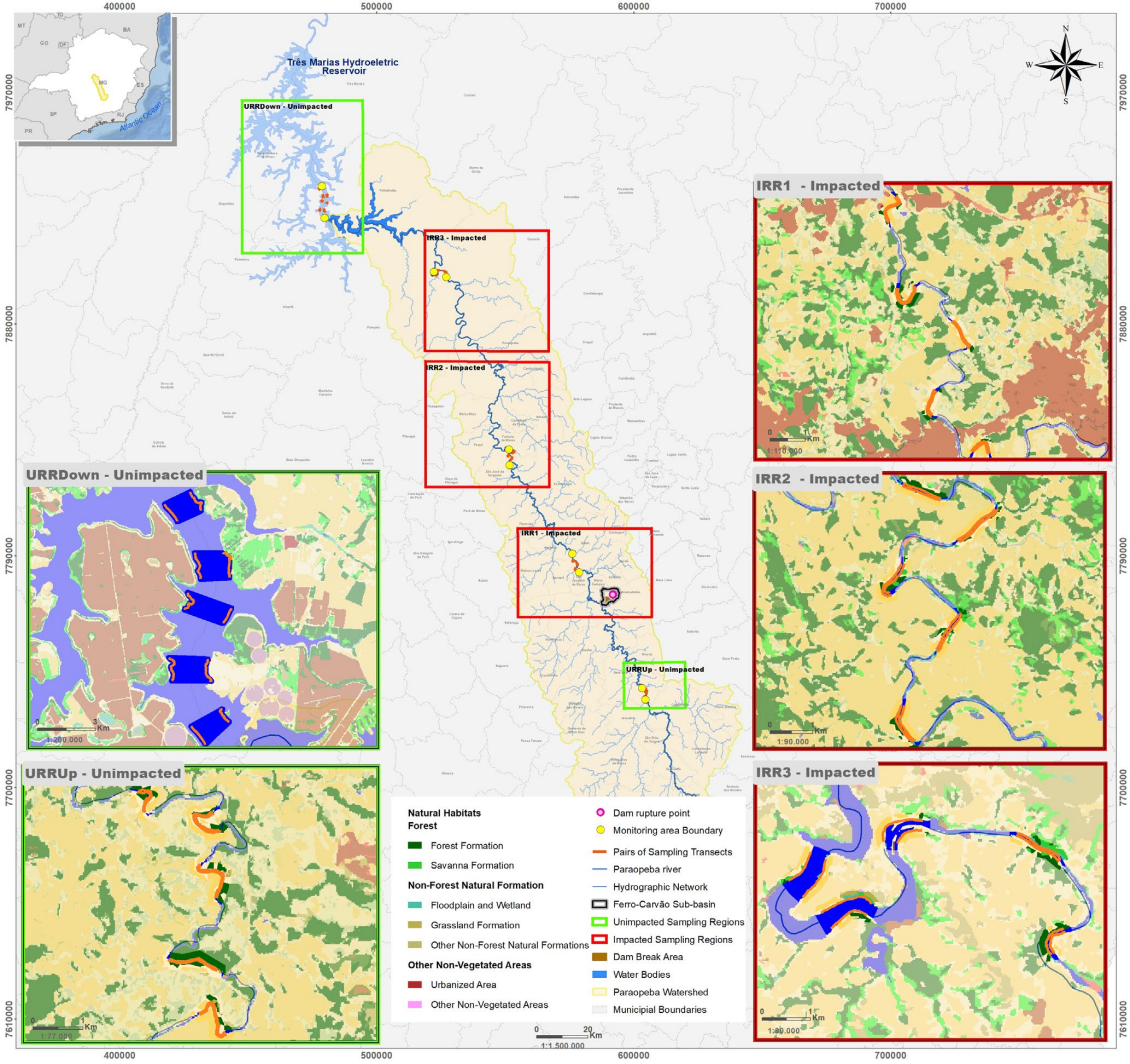
Sampling regions and transects surveyed along the Paraopeba river, Minas Gerais, Brazil, for the assessment of *L. longicaudis* occupancy dynamics following the 2019 Brumadinho tailings‐dam collapse. The river was divided into five regions according to impact context: Upstream unimpacted river reaches (URRUp), downstream unimpacted reaches within the Três Marias Hydroelectric Reservoir (URRDown), and three impacted reaches downstream of the Ferro‐Carvão–Paraopeba confluence (IRR1–IRR3). Insets show detailed views of each region, with transects (orange lines) and major land‐cover classes.

We defined five distinct regions based on their impact context, encompassing both unimpacted river reaches (URR) and impacted river reaches (IRR). These regions represent different longitudinal positions along the Paraopeba river and are separated by at least 50 km. The URR includes the upstream region located beyond the influence zone (URRUp) and the region within the Três Marias Hydroelectric Power Plant reservoir (URRDown), which was downstream of the Ferro‐Carvão confluence but considered unaffected because tailings did not reach the reservoir (Laureano et al. 2022; Kobayashi et al. 2023; Rojas‐Aguirre and Garcia 2025). Conversely, the IRR comprises downstream segments of the Ferro‐Carvão–Paraopeba confluence (IRR1, IRR2, and IRR3), empirically documented as exposed to tailings (Figure 1).

Within each of these five regions, the Paraopeba river was subdivided into 15‐km segments, from which one segment was randomly selected to represent that region. Thus, the effective study area consisted of five discrete 15 km river stretches distributed along the river's longitudinal gradient. In each selected segment, we systematically established ten 1‐km transects—five on each bank—with a minimum spacing of two kilometers between adjacent transects. To ensure spatial alignment between opposite‐bank transects, mirrored geographic coordinates were applied.

### 2.2 Sampling Design and Field Protocol

Fieldwork was conducted during 16 sampling campaigns between January 2021 and February 2025, all during the rainy period to maximize navigability and access to transects. Importantly, field campaigns were organized into temporal seasons, which constituted the primary temporal unit of analysis and did not correspond to calendar years. Specifically, each season encompassed survey campaigns conducted from the end of one year through the beginning of the following year, deliberately spanning the rainy period and associated hydrological conditions. Initially, until the sixth campaign (the first two seasons), each transect was sampled on two distinct days within each campaign. However, a preliminary analysis of data from these six campaigns revealed that the second consecutive visit to the same transects did not significantly alter the detection status of otters compared to the first visit within each campaign, with variation observed in only 6% of the revisits. Based on this finding, from the third season (seventh campaign) onwards, the methodology was adjusted to a single daily sampling per transect/region per campaign. Additionally, the number of campaigns per season was increased from three to four to provide more temporal replicates within each season, as recommended by MacKenzie et al. (2003) for multi‐season occupancy analyses (see data analysis for details). Consequently, the first two sampling seasons each comprised three campaigns, while subsequent seasons consisted of four campaigns each.

We surveyed transects from an aluminum boat equipped with a 15‐hp motor, traveling at speeds not exceeding 7 km/h. Each river stretch and its corresponding bank were meticulously inspected for both direct and indirect signs of neotropical otter presence. Where river banks permitted, we conducted ground searches for tracks, holts, couches, spraints, and anal jelly (mucus) (Pardini and Trajano 1999; Kasper et al. 2008). Upon locating tracks, spraints, and anal jelly, we removed them to avoid double‐counting in subsequent surveys (Santos and Reis 2012). Additionally, we monitored the status of holts and couches across campaigns to assess their continued use, ensuring accurate representation of otter occupancy over time.

We classified spraints as recent (moist) or old (dry) and recorded the presence or absence of anal jelly. Each spraint was assigned to one of seven substrate categories: grass, bare soil, bank rock, in‐water rock, fallen log/wood in the water, fallen log/wood on the bank, and sand/soil (Santos and Reis 2012). Deposition sites were identified as either latrines (open areas repeatedly used for sprainting) or shelters (covered/protected environments) (Kasper et al. 2008). Dens (holts) were categorized into three types: cavities among tree roots, cavities among rocks, and cavities within dense vegetation (Pardini and Trajano 1999; Santos and Reis 2012). We also recorded whether neotropical otters excavated the dens or used pre‐existing natural cavities (Kasper et al. 2008).

However, the same individual could have left different types of evidence (e.g., spraints, tracks, and holts). In such cases, when two or more pieces of evidence were obtained in the same transect during the same sampling campaign, they were counted as a single independent record.

### 2.3 Modeling Initial Occupancy, Colonization, Extinction Probabilities, and Detection of *L. longicaudis* in Relation to Predictor Variables

Occupancy probability (Ψ) represents the probability that a sample unit i is occupied by a given species. Detection probability (*p*), on the other hand, refers to the chance of recording the species during a specific sampling occasion *j* (i.e., field campaign in our study) within sample unit *i* (i.e., transect pair in our study), assuming the species is indeed present in the area (MacKenzie et al. 2002). Changes in the species' occupancy status over time (i.e., sampling seasons) are influenced by dynamic parameters related to colonization and extinction processes within the sample units (MacKenzie et al. 2003).

The initial occupancy probability of otters (Ψ*ᵢ* in the first sampling season) can change over subsequent seasons due to variables influencing colonization and extinction processes within sample units (MacKenzie et al. 2003). Colonization (*γ*) is the probability that an unoccupied sample unit at time *t* becomes occupied by the species at time *t* + 1 (MacKenzie et al. 2003). Extinction (*ε*) is the probability that an occupied sample unit at time *t* becomes unoccupied by the species at time *t* + 1 (MacKenzie et al. 2003). All parameters (Ψ*ᵢ*, *γ*, *ε*, and *p*) can be modeled as functions of predictor variables.

Occupancy analyses were evaluated according to five a priori ecological hypotheses for the parameters Ψᵢ, *γ*, and *ε*. These hypotheses were related to spatial, temporal, and environmental gradients potentially associated with the dam collapse, including distance to the confluence of the Ferro‐Carvão stream with the Paraopeba river, time since the dam collapse (i.e., from the first to the fifth sampling season), water flow velocity, turbidity, and proportion of natural areas (Appendix 1).

Detection probability (*p*) was assessed based on time since the dam collapse and distance to the confluence. In addition, we accounted for variation in sampling effort among seasons. The first two sampling seasons included fewer field campaigns (*N* = 3) whereas later seasons included four campaigns, and the final season was restricted to two campaigns due to logistical and access constraints. To account for this variation, we used the number of campaigns per season as a predictor variable for detection probability (*p*).

Overall, our modeling framework was explicitly hypothesis‐driven. Initial occupancy (ψ_*i*_) was modeled as a function of spatial gradients related to the dam collapse and habitat structure, reflecting baseline patterns of site use along the river. Colonization (*γ*) and extinction (*ε*) probabilities were modeled using the same set of spatial and environmental variables to evaluate whether post‐disturbance occupancy dynamics varied along these gradients over time. Detection probability (*p*) was modeled using temporal, spatial and survey‐specific variables expected to influence sign detectability, thereby ensuring a clear separation between the ecological and observation processes.

The longitudinal position of each transect pair was determined relative to the confluence of the Ferro‐Carvão stream with the Paraopeba river. For this measurement, we utilized Google Earth Pro (version 7), based on high‐resolution Maxar satellite imagery from 2021 (Google 2022). The distances of the transects in URRUp were recorded as negative, indicating their location upstream of the aforementioned confluence.

To assess the temporal effect of the dam collapse on otter occupancy probability, we modeled the parameters *γ* (colonization probability) and ε (extinction probability) as functions of a continuous variable simulating a “trend effect.” This approach allowed for the linear change of otter colonization and extinction probabilities across transect pairs over the sampling seasons. Similarly, this method was applied to model detection probability.

Water flow velocity and turbidity were measured using a flowmeter and a turbidimeter, respectively. Measurements were taken at three equidistant points (beginning, middle, and end) of each transect. During data collection, the boat was stationary with the engine idling to maintain a fixed position while acquiring data. This stationary data collection protocol was standardized and consistently applied across all field campaigns. For each campaign and sample unit, the average values of water flow velocity and turbidity were calculated within each sampling season. The average value from the first sampling season for each variable was used as a predictor variable to model the initial occupancy parameter (Ψ*ᵢ*), while the average values from subsequent seasons were used to model the colonization (*γ*) and extinction (*ε*) parameters.

Land use and land cover assessment in the study area was conducted using the MapBiomas database (Mapbiomas 2025) on Google Earth Engine. This database was instrumental in mapping and spatially characterizing the proportion (%) of natural habitats (comprising forest formations, savanna formations, grasslands, and wetlands) within 150‐m buffers around each transect (considering only the external part of the buffers, since the internal part consists only of water). The choice of a 150‐m buffer is based on the distance that *L. longicaudis* typically travels to the surrounding riverbanks in areas where they occur (Bertonatti and Parera 1994). The average of this proportion over the seasons of data collection and for each transect pair was used to model the parameters Ψ*ᵢ*, *γ*, and *ε*.

### 2.4 Data Analysis

Direct and indirect records of *L. longicaudis* occurrence were grouped by field campaign within each sampling season, generating detection histories for each sampling unit (i.e., pairs of transects). For each campaign (i.e., secondary sampling occasion) within each season (i.e., primary sampling occasion), the species was recorded as detected (1) or not detected (0), following the approach proposed by MacKenzie et al. (2003). From the detection history matrix, we used a dynamic or “multi‐season” occupancy model (MacKenzie et al. 2003) in the *unmarked* package (Fiske and Chandler 2011) in R (R Core Team 2023) to analyze the data. This model assumes occupancy status remains constant across campaigns within the same season (i.e., population closure), but allows occupancy status to change between seasons. Because the sampling period within each season was short and always conducted during the rainy season, we assumed population closure within each season.

A set of models was constructed based on our a priori hypotheses about factors likely to influence the parameters initial occupancy (Ψ*ᵢ*), colonization (*γ*), extinction (*ε*), and detection (*p*) of the species. We also included null models (e.g., Ψ*ᵢ*(·) *γ*(·) *ε*(·) *p*(·)), which include only the intercepts, with no predictor variables associated with the parameters. Specifically, candidate models were constructed to represent alternative ecological hypotheses regarding spatial disturbance effects, habitat quality, and temporal dynamics.

Using the Akaike Information Criterion corrected for small sample sizes (AICc), we considered models with ΔAICc ≤ 2 as equally plausible given the data (Burnham and Anderson 2002). First, we assessed potential overdispersion in the data (i.e., lack of independence in detection histories across sampling units) using the goodness‐of‐fit test proposed by MacKenzie and Bailey (2004). This test evaluates, through simulations, whether the variance inflation factor (*ĉ*) is greater than 1 with a *p*‐value ≤ 0.05. Values of *ĉ* > 1 and *p* ≤ 0.05 indicate overdispersion, in which case model ranking should be adjusted using the estimated ĉ, and results interpreted with QAICc, which follows the same theoretical framework as AICc (Burnham and Anderson 2002). In this study, based on 10,000 simulations, the test did not reveal overdispersion (*χ* ^2^ = 35.11; *ĉ* = 1.23; *p* = 0.17). Therefore, we used AICc for model ranking and result interpretation. The test was performed using the “AICcmodavg” package (Mazerolle 2020) in R (R Core Team 2023).

Maximum‐likelihood methods implemented in the *unmarked* package were used to obtain estimates of occupancy, colonization, extinction, and detection of *L. longicaudis* across sampling seasons and sampling units (MacKenzie et al. 2003). For example, suppose a detection history in the first sampling season for a given sampling unit (the pair of transects) in which the otter occupies that unit and is detected (through either direct or indirect signs) in the first and last of the three occasions (field campaigns) in the first season. We can represent that history as 101, where 1 indicates the species was recorded and 0 that it was not. In the second season, there are no detections in any of the three occasions (000). Thus, such a hypothetical history can be represented by *h*_1_ = 101,000. From this hypothetical history, one can infer that from one sampling season to the next, either the otter continues occupying the sampling unit but was not detected in the second season, or else the species ceased to occupy the sampling unit in the second season. Mathematically, this can be translated as:

$$\begin{array}{c} \text{Probability}\left(h_{1}=101,000\right)=\psi_{i}p_{1,1}\left(1-p_{1,2}\right)\\ \;p_{1,3}\left\{\left(1-\varepsilon_{1}\right)\prod_{j=1}^{3}\left(1-p_{2,j}\right)+\varepsilon_{1}\;\right\} \end{array}$$

As proposed by MacKenzie et al. (2003), the likelihood of each model was calculated according to the equation below, which accounts for all detection histories of the otter across sampling seasons and occasions. The parameter values that maximize the likelihood were obtained by:

$$L\left(\psi_{i},\varepsilon ,\gamma ,p\;|\;h_{1},..\ldots ,h_{N}\right)=\prod_{i=1}^{N}\text{Probability}\left(h_{i}\right)$$

Occupancy in subsequent seasons was estimated based on the updating equation proposed by MacKenzie et al. (2003):

$$\Psi_{t+1}=\Psi_{t}\;\left(1-\varepsilon_{t}\right)+\left(1-\Psi_{t}\right)\;\gamma_{t}$$

All predictor variables were standardized prior to model construction as $Z=\frac{\mathrm{Xi}-\mu }{\sigma }$, where *Z* is the standardized variable, and *X*_*i*_, *μ*, and *σ* represent a given value, the mean, and the standard deviation of the original variable *X*, respectively.

## 3 Results

A total of 1094 km was surveyed across the five monitoring seasons. Overall, 286 signs of otter presence were recorded, including 283 indirect signs and three visual observations (Appendices 2 and 3). Among the indirect signs, tracks were the most frequent (*N* = 173), followed by spraints (*N* = 97) and holts (*N* = 42), all located in natural cavities among tree roots. The highest recording frequencies were observed in IRR1, IRR2, and IRR3, whereas URRUp and URRDown consistently exhibited the lowest numbers of records across all seasons (Appendix 2).

Spraints were classified as dry (i.e., without mucus; *N* = 50) or fresh with mucus (*N* = 47). Deposition sites varied, with most spraints found on soil/sand (*N* = 48), followed by fallen logs/wood in the water (*N* = 13), fallen logs/wood on the bank (*N* = 12), bare soil (*N* = 11), rocks on the bank (*N* = 9), and rocks in the water (*N* = 4). Spraints were more frequently deposited in latrines (*N* = 83) than in shelters (*N* = 14).

**FIGURE 2:**
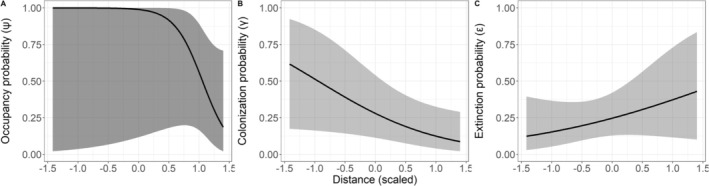
Initial occupancy (A), colonization (B), and extinction (C) of *L. longicaudis* as a function of distance from the confluence of the Ferro‐Carvão stream with the Paraopeba river (±95% CI). These estimates are based on the most parsimonious model (ΔAICc ≤ 2). Distance to the confluence was standardized (centered and scaled) prior to analysis to improve parameter estimation and comparability among variables.

Among the candidate occupancy models, a single parsimonious model had high support (i.e., ΔAICc < 2; Table 1). This model indicated that occupancy probability in the first sampling season, as well as colonization and extinction probabilities between seasons, were influenced by the distance from the confluence of the Ferro‐Carvão stream with the Paraopeba river. No other variable affected these parameters (i.e., ΔAICc > 2; Table 1). However, contrary to our expectations, occupancy probability declined with increasing distance from the Ferro‐Carvão–Paraopeba confluence (*β* = −4.30 ± 2.89 SE; Figure 2A). Colonization probability likewise decreased with distance (*β* = −1.00 ± 0.50 SE; Figure 2B), whereas extinction probability increased with distance (*β* = 0.60 ± 0.56 SE; Figure 2C).

**TABLE 1:** Ten highest‐ranked candidate models used to model initial occupancy (Ψ*ᵢ*), colonization (*γ*), extinction (*ε*), and detection (*p*) of *L. longicaudis* .

Model	AICc	ΔAICc	C*w*	*K*
Ψ_*i*_(Distance) + *γ*(Distance) + *ε*(Distance) + *p*(.)	420.75	0.00	0.34	7
Ψ_*i*_ (Vegetation) + *γ*(Vegetation) + *ε*(Vegetation) + *p*(.)	423.22	2.46	0.44	7
Ψ_*i*_(Distance) + *γ*(Distance) + *ε*(Distance) + *p*(Effort)	423.66	2.90	0.52	8
Ψ_*i*_(.) + *γ*(.) + *ε*(.) + *p*(.)	423.84	3.09	0.59	4
Ψ_*i*_(.) + *γ*(.) + *ε*(.) + *p*(Effort)	424.25	3.49	0.70	5
Ψ_*i*_(Vegetation) + *γ*(Vegetation) + *ε*(Vegetation) + *p*(Effort)	424.50	3.75	0.75	8
Ψ_*i*_(Distance) + *γ*(Distance) + *ε*(Distance) + *p*(Distance)	425.03	4.27	0.80	8
Ψ_*i*_(Velocity) + *γ*(Velocity) + *ε*(Velocity) + *p*(.)	425.36	4.61	0.83	7
Ψ_*i*_(Distance) + *γ*(Distance) + *ε*(Distance) + *p*(Time)	425.79	5.03	0.86	8
Ψ_*i*_(.) + *γ*(.) + *ε*(.) + *p*(Distance)	425.94	5.18	0.89	5

*Note:* Distance = distance to the confluence of the Ferro‐Carvão stream with the Paraopeba river; Time = sampling seasons (time since the dam collapse); Velocity = water flow velocity in each river section; Turbidity = water turbidity in each river section; Vegetation = the proportion (%) of natural habitats; Effort = the number of campaigns per season; “.” = intercept only structure; C*w* = cumulative AICc Weight; *K* = number of parameters.

**FIGURE 3:**
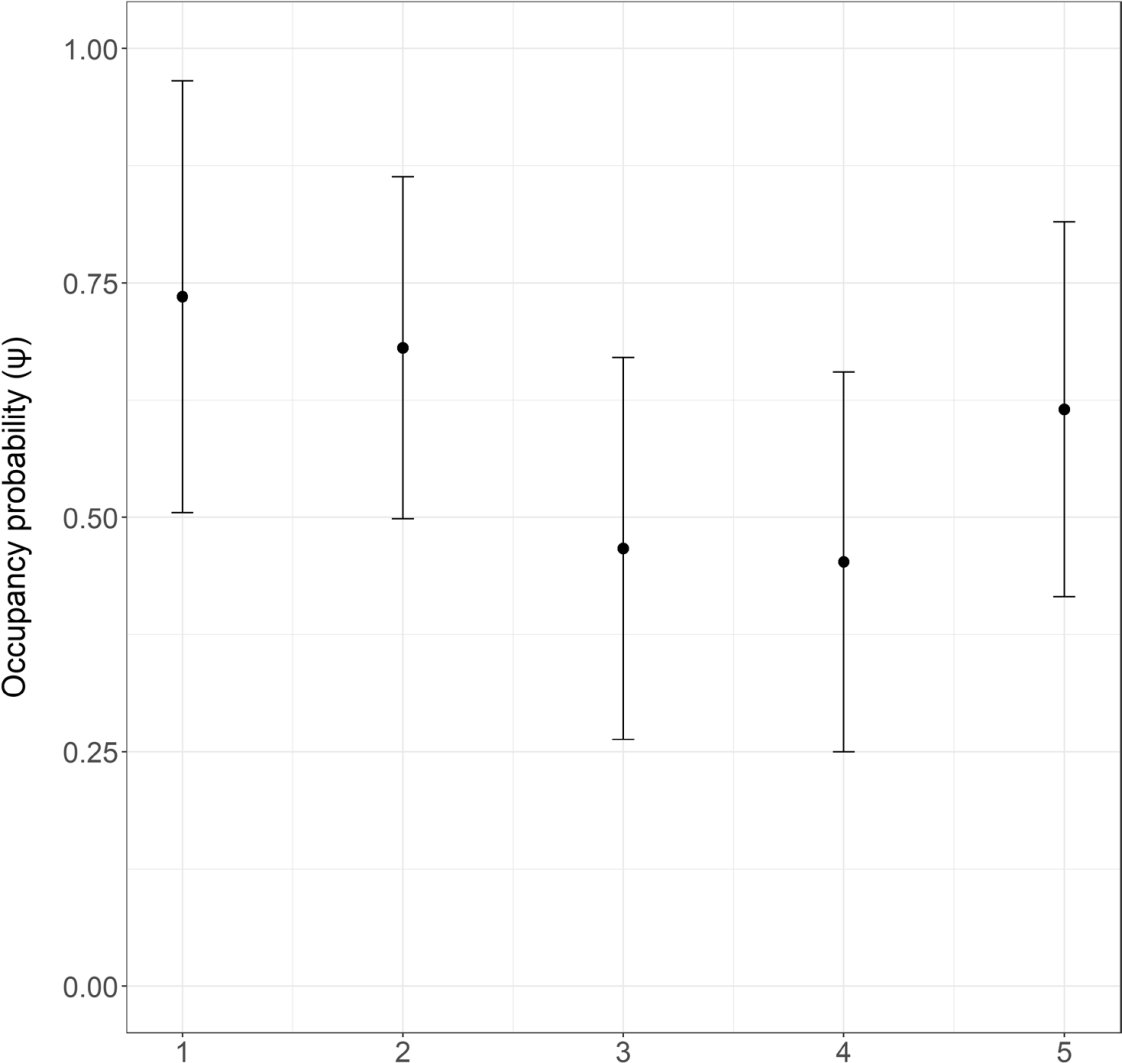
Mean occupancy probability (±95% CI) of *L. longicaudis* among sampling seasons. These estimates are based on the single most parsimonious model (Δ AICc ≤ 2).

**FIGURE 4:**
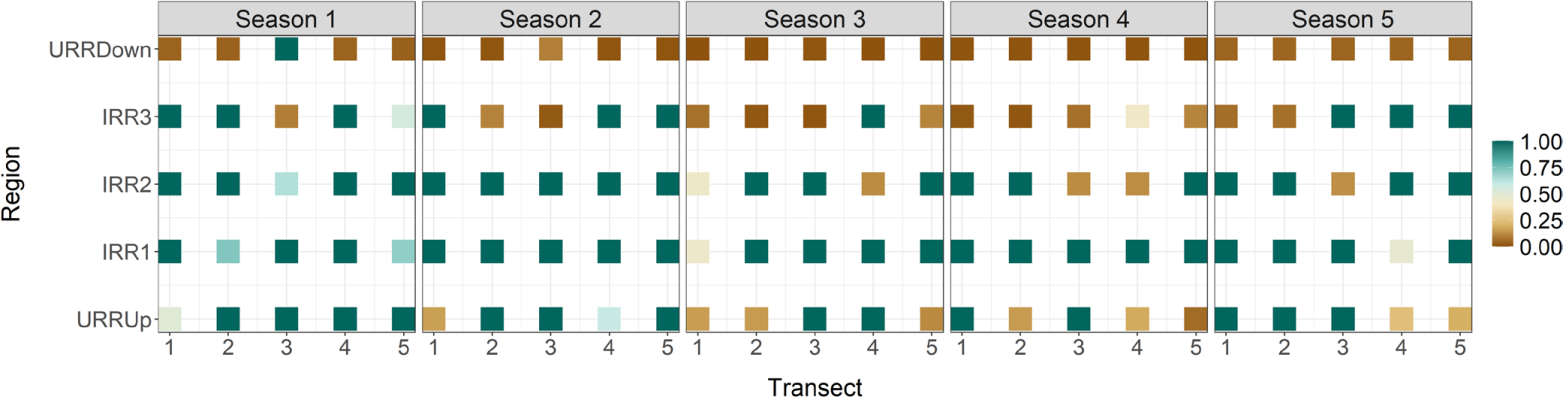
Occupancy probability of *L. longicaudis* estimated from the top‐ranked model (ΔAICc ≤ 2) for each pair of transects by region and sampling season. The Paraopeba river was divided into five regions according to impact from the dam collapse: URRUp—unimpacted reach upstream of the Ferro‐Carvão–Paraopeba confluence; IRR1—impacted reach extending from Casa Branca (immediately upstream of the Ferro‐Carvão confluence) to downstream of Brumadinho; IRR2 and IRR3—impacted reaches downstream of Brumadinho and upstream of the Retiro Baixo reservoir; URRDown—unimpacted reach within the Três Marias reservoir.

Across sampling seasons, overall occupancy declined, with a rebound in the final season (Figure 3). Transect‐level estimates indicate that URRUp declined slightly until season 4 and then increased in season 5 (Figure 4). Occupancy remained high across all transects in IRR1 (Figure 4). Regions IRR2 and IRR3 showed similar, fluctuating dynamics over time, whereas URRDown exhibited consistently low occupancy throughout the study (Figure 4).

Finally, none of the variables analyzed influenced the detection probability (*p*) of *L. longicaudis* (Table 1), which was ~0.49 ± 0.04 SE.

## 4 Discussion

This study addresses a key knowledge gap by examining post‐disturbance occupancy dynamics of *L. longicaudis* following a tailings‐dam collapse. Although anthropogenic threats such as habitat degradation, pollution, hunting, and mining have been extensively investigated in aquatic and semi‐aquatic mammals (Navarro‐Picado et al. 2017; Smith et al. 2020; Brum et al. 2021; Sanders et al. 2023; Rosas‐Ribeiro et al. 2024; Crockett et al. 2025), assessments of post‐disturbance, large‐scale events like dam failures are relatively new features that remain scarce. Understanding these disturbances is essential to reveal the mechanisms by which environmental disasters can alter population dynamics and to inform effective conservation and management strategies.

Our multi‐season analysis reveals continuous distribution and high occupancy probabilities of *L. longicaudis* in the Paraopeba river, including impacted reaches, after the Brumadinho dam collapse. Contrary to our initial expectation that sites closer to the Ferro‐Carvão–Paraopeba confluence (i.e., smaller distance) would exhibit reduced occupancy and colonization, as well as elevated extinction due to greater tailings exposure, the top‐ranked model revealed the opposite pattern: occupancy and colonization decreased with increasing distance from the confluence, while extinction increased with distance. Consistent with these model results, recording frequencies were highest in IRR1 (the closest reach from the impacted area, extending from Casa Branca, immediately upstream of the Ferro‐Carvão confluence), and in the impacted reaches IRR2 and IRR3 (downstream of Brumadinho and upstream of the Retiro Baixo reservoir). In contrast, URRUp (the unimpacted reach upstream of the confluence) and URRDown (the unimpacted reach within the Três Marias reservoir) showed the lowest numbers of records across all seasons.

Therefore, otter occupancy dynamics were not lower at sites closer to the Ferro‐Carvão confluence, suggesting that, within the temporal window evaluated, occupancy, colonization, and extinction probabilities were not strongly associated with proximity to the dam collapse. This finding reinforces the hypothesis that *L. longicaudis* exhibits ecological flexibility and tolerance to some anthropogenic environmental changes (Coletti et al. 2013; Krug et al. 2019). A previous study (Lanna et al. 2022) likewise reported higher numbers of otter occurrences in this same area. The elevated recording frequencies we observed in impacted reaches may reflect greater availability of sandbanks, which may facilitate track detection. Notably, none of the predictor variables we evaluated, including distance (which is correlated with region), affected detection probability, indicating that the spatial pattern of records is not readily explained by the detection covariates included in our models.

In reaches such as those sampled within the Três Marias hydroelectric reservoir (URRDown), we recorded only two occurrences (both restricted to the first sampling season). Although *L. longicaudis* has been recorded in artificial reservoirs (Santos and Reis 2012), use of impoundments appears less intensive, likely due to shoreline characteristics such as increased disturbance, reduced riparian vegetation, and the absence of natural banks; these features are consistent with lower occupancy and detectability (Quadros 2012; Pérez et al. 2020). Reservoir shores typically offer limited native riparian cover necessary for refuge (Kasper et al. 2008) and are often subject to anthropogenic impacts (Linares et al. 2020), factors that likely discourage otter use. The absence of an effect of native vegetation variation along the river on occupancy dynamics may indicate that such relationships occur at finer spatial scales or under specific habitat conditions not captured by our regional‐scale analysis.

The other environmental variables evaluated did not influence occupancy, colonization, or extinction probabilities in the sampled stretches of the Paraopeba river. Although previous studies have shown that *L. longicaudis* tends to prefer clearer waters, likely related to foraging efficiency (Cruz García et al. 2017; de Almeida and Pereira 2017), turbidity was not deemed as a determining factor for its occurrence in our study.

Similarly, variation in mean water flow velocity did not influence otter occupancy, even though a preference for faster flows is generally attributed to higher concentrations of dissolved oxygen, crucial for fish that make up much of the otter's diet as well as to the creation of microhabitats favorable to specific prey, including the dislodgement of invertebrates that facilitates fish predation (Larivière 1999). However, the Paraopeba river generally has a high flow, and it is unlikely that the range of flow fluctuations observed in our study reduced the availability of potential otter prey, given the interdependence between water flow, physicochemical parameters, and primary productivity in river systems (Cruz García et al. 2017; Bernhardt et al. 2022).

Occupancy probability showed an overall stability throughout the study areas and period, despite the influence of distance on occupancy dynamic parameters. Although a slight general decline was detected in populations during seasons three and four, it increased again in season five, with confidence intervals overlapping the initial occupancy estimate. This pattern indicates consistent use of the region by otters over the sampled years. Additionally, the observations from this study, consistent with previous studies in southern Brazil, indicate a preference of *L. longicaudis* for shelters under tree roots or along riverbanks (Kasper et al. 2004, 2008). This pattern may reflect both an intrinsic preference of the species and the greater availability of such shelter types compared to rock cavities along the sampled reaches.

Spraints were predominantly recorded in open latrines, mainly on soil or sand substrates, suggesting a tendency toward social communication near the Paraopeba river margins. Otters use scent marks, such as spraints and secretions, for intraspecific communication, with variations in marking locations reflecting different behavioral strategies (Larivière 1999). While some studies have suggested a greater deposition of spraints inside shelters (Quadros and Monteiro‐Filho 2002; Kasper et al. 2004), other research has also documented marking in visible sites such as stones and fallen logs along river margins (Pardini and Trajano 1999; Quadros and Monteiro‐Filho 2002; Kasper et al. 2008), reinforcing the idea that spraints are used as a means of intraspecific communication along riverbanks.

Overall, our study enhances understanding of post‐disturbance habitat use by *L. longicaudis* following the B1 dam collapse, showing that otters maintain a continuous distribution and relatively stable occupancy probability throughout the monitored seasons from 2021 to 2025. These findings underscore a persistence of the *L. longicaudis* populations, despite the large‐scale anthropogenic disturbances faced by the region, including the mining dam collapse. The persistence of otters across impacted and unimpacted stretches of the Paraopeba river indicates that the species can continue to exploit available habitats and resources despite environmental disturbances. Notably, occupancy dynamics did not show strong declines near the Ferro‐Carvão confluence, the most affected area, suggesting tailings deposition was not associated with persistent barriers to habitat use or colonization during the study period. Furthermore, spatial differences in occupancy, particularly the lower otter occurrence in hydroelectric reservoir stretches, emphasize the influence of habitat structure (e.g., reduced riparian cover, altered shoreline morphology) on species distribution and habitat suitability.

It is also worth noting that because monitoring started 2 years after the B1 dam collapse, our results should be interpreted as reflecting post‐disturbance habitat use rather than immediate impacts or acute responses to the disaster. It is therefore possible that neotropical otters had already recolonized or adjusted their spatial use prior to the onset of our surveys. Consequently, patterns of occupancy persistence and stability observed in this study may reflect spatial resilience or early recovery of habitat use, rather than an absence of disturbance effects. In addition, although we did not explicitly assess water pollution or prey availability, the continued occurrence and occupancy of neotropical otters suggest that key ecological requirements were met in these river stretches during the study period. As fish constitute the primary component of the species' diet, otter occurrence may indirectly indicate the persistence or recovery of fish populations. Variables such as turbidity and riparian natural cover, included in our models, likely captured part of the spatial variation in water condition and habitat quality. Similarly, distance from the collapse may integrate multiple disturbance‐related gradients that were not explicitly measured.

Our inferences are also conditioned on the assumptions inherent to dynamic occupancy models. These include closure within each sampling season, such that site occupancy status does not change during repeated surveys, and a detection process that is adequately described by the covariates included in the model (MacKenzie et al. 2003). Given the high mobility of *L. longicaudis* , temporary movements in and out of sampling units within seasons cannot be fully ruled out. However, the relatively short duration of repeated surveys within each season and the use of spatially defined sampling units reduce the likelihood that such movements substantially biased occupancy estimates. Additionally, although we explicitly tested for overdispersion and found no evidence of extra‐binomial variation, some degree of spatial dependence among nearby sampling units may persist. Our results should therefore be interpreted as reflecting species‐level patterns of habitat use rather than individual‐level space use. Finally, the use of environmental proxies, such as distance from the dam collapse, turbidity, and proportion of natural riparian cover, represents a necessary simplification of complex ecological processes. While these variables capture key gradients relevant to post‐disturbance habitat conditions, unmeasured factors such as fine‐scale habitat structure, prey availability, and water chemistry may also influence occupancy dynamics.

Given these patterns, continued long‐term monitoring is crucial to detect potential delayed or cumulative effects, such as those linked to bioaccumulation of metals or progressive changes in prey availability. Investigating physiological biomarkers and reproductive success over time will be essential for understanding whether chronic sublethal effects emerge. Moreover, conservation strategies should prioritize the maintenance and restoration of riparian vegetation, the reduction of anthropogenic pressures (e.g., sewage input, habitat modification), and the management of reservoir habitats to improve connectivity and ecological quality. Such measures will not only support the persistence of *L. longicaudis* but also enhance the overall resilience and integrity of freshwater ecosystems in the Paraopeba basin.

## Author Contributions

**Rodrigo Lima Massara:** conceptualization (equal), data curation (equal), formal analysis (lead), investigation (equal), methodology (equal), validation (equal), visualization (equal), writing – original draft (lead), writing – review and editing (lead). **Paloma Marques Santos:** data curation (equal), formal analysis (supporting), software (lead), validation (equal), visualization (equal), writing – review and editing (equal). **Rodolfo Stumpp:** conceptualization (equal), data curation (equal), formal analysis (equal), investigation (equal), methodology (equal), project administration (equal), validation (equal), visualization (equal), writing – review and editing (equal). **Aline Saturnino Costa:** conceptualization (equal), data curation (equal), methodology (equal), project administration (equal), validation (equal), writing – review and editing (equal). **Joyce Ramos Rodrigues:** conceptualization (equal), data curation (equal), validation (equal), visualization (equal), writing – review and editing (equal). **Ana Yoko Ykeuti Meiga:** conceptualization (equal), methodology (equal), project administration (equal), validation (equal), writing – review and editing (equal). **Ricardo R. C. Solar:** conceptualization (equal), funding acquisition (equal), project administration (equal), writing – review and editing (equal). **Mariana Neves Moura:** conceptualization (equal), funding acquisition (equal), methodology (equal), project administration (equal), resources (equal), validation (equal), writing – review and editing (equal). **Tiago Dornas:** funding acquisition (equal), methodology (equal), project administration (equal), validation (equal), writing – review and editing (equal). **Cristiane Cäsar:** funding acquisition (equal), project administration (equal), validation (equal), writing – review and editing (equal). **Adriano Paglia:** conceptualization (equal), formal analysis (equal), funding acquisition (equal), investigation (equal), methodology (equal), project administration (equal), supervision (equal), validation (equal), visualization (equal), writing – review and editing (equal).

## Funding

This work was supported by VALE S.A.

## Disclosure

This product is part of the Damage Assessment Programme on Biotic Environment (PDD), developed, coordinated, and managed by Amplo Engenharia e Gestão de Projetos and the Federal University of Minas Gerais (UFMG), and funded by Vale S.A. as part of the company's obligations to repair damages caused by the collapse of the B1 dam. The program is conducted in accordance with requirements established by environmental agencies and is audited and overseen by the State Forestry Institute (IEF) and the Public Prosecutor's Office of Minas Gerais (MPMG). C.C. is employed by Vale S.A. and declares that the company had no influence over the decision to publish this work, nor over its technical or scientific content. All authors affirm that this article exclusively reflects the results of research conducted in accordance with recognized scientific standards, and that the conclusions represent the authors' shared interpretation based on the data collected and relevant literature. Field surveys were conducted under collection permits n° 39548342/2021/GCSIL‐IEF, n° 45894234/2022/GCSIL‐IEF, n° 57109843/2022/GCSIL‐IEF, n° 74444352/2023/GCSIL‐IEF, n° 77911947/2023/GCSIL‐IEF, and n° 100443850/2024/GCSIL‐IEF, issued by the State Forestry Institute of Minas Gerais (IEF). This is product number PDD‐0016 in the PDD publication series.

## Conflicts of Interest

The authors declare no conflicts of interest.

## Supporting information


**Data S1:** ece373366‐sup‐0001‐Supinfo.zip.

## Data Availability

All data supporting the findings of this study are available within the main text, Supporting Information, and appendices.

## Appendix 1

Mean, minimum, and maximum values of predictor variables evaluated as potential hypotheses for initial occupancy (Ψ_*i*_), colonization (*γ*), extinction (*ε*), and detection (*p*) probabilities of *L. longicaudis* along the Paraopeba river. Summary statistics were computed at the scale of the sampling unit (pairs of transects) across all study sites. Site‐level predictors include distance to the Ferro‐Carvão confluence and proportion of natural riparian area. For time‐varying predictors (turbidity and water flow velocity), mean, minimum, and maximum values are reported for each sampling season. Occupancy dynamics (Ψ*ᵢ*, *γ*, and *ε*) were evaluated in relation to distance to the confluence, time since the dam collapse, water flow velocity, turbidity, and proportion of natural areas, while detection probability (*p*) was assessed as a function of time since the dam collapse and distance to the confluence.

**Table:**

Variable	Mean	Minimum value	Maximum value
Distance to the confluence of the Ferro‐Carvão stream with the Paraopeba river (m)	143,354.43	30,287.58	379,371.67
Water velocity (m/s)—season 1	0.23	0.08	0.52
Water velocity (m/s)—season 2	0.38	0.07	0.91
Water velocity (m/s)—season 3	0.46	0.12	0.91
Water velocity (m/s)—season 4	0.43	0.12	0.85
Water velocity (m/s)—season 5	0.32	0.18	0.50
Turbidity—season 1 (NTU)	83.19	1.83	244.64
Turbidity—season 2 (NTU)	86.12	16.30	155.57
Turbidity—season 3 (NTU)	295.55	35.52	507.23
Turbidity—season 4 (NTU)	262.45	37.56	592.08
Turbidity—season 5 (NTU)	118.02	66.18	184.57
Proportion (%) of natural area	19.93	0.00	58.64

## Appendix 2

Type and number of *L. longicaudis* presence records across five monitoring seasons in regions of the Paraopeba river. The Paraopeba river was divided into five regions according to impact from the dam collapse: URRUp—unimpacted reach upstream of the Ferro‐Carvão–Paraopeba confluence; IRR1—impacted reach extending from Casa Branca (immediately upstream of the Ferro‐Carvão confluence) to downstream of Brumadinho; IRR2 and IRR3—impacted reaches downstream of Brumadinho and upstream of the Retiro Baixo reservoir; URRDown—unimpacted reach within the Três Marias reservoir.

**Table:**

Season	Region	Spraints	Tracks	Holts	Visualizations	Total of registers
1 (campaign 1: 2021‐01‐18 to 2021‐02‐06; campaign 2: 2021‐02‐22 to 2022‐03‐15; campaign 3: 2021‐04‐19 to 2021‐05‐06)	URRUp	2	10	0	0	12
	IRR1	10	26	5	1	42
	IRR2	7	23	3	0	33
	IRR3	6	19	0	0	25
	URRDown	2	0	0	0	2
Total of registers (season 1)		27	78	8	1	114
2 (campaign 4: 2021‐11‐29 to 2021‐12‐13; campaign 5: 2022‐01‐27 to 2022‐02‐07; campaign 6: 2022‐04‐19 to 2022‐05‐05)	URRUp	3	4	0	0	7
	IRR1	12	35	4	0	51
	IRR2	7	21	2	0	30
	IRR3	7	4	0	1	12
	URRDown	0	0	0	0	0
Total of registers (seasons 2)		29	64	6	1	100
3 (campaign 7: 2022‐11‐28 to 2022‐12‐15; campaign 8: 2023‐01‐16 to 2023‐01‐23; campaign 9: 2023‐02‐06 to 2023‐02‐11; campaign 10: 2023‐03‐13 to 2023‐03‐18)	URRUp	1	1	0	0	2
	IRR1	5	4	6	0	15
	IRR2	3	9	3	0	15
	IRR3	2	0	0	0	2
	URRDown	0	1	0	0	0
Total of registers (season 3)		11	14	3	0	34
4 (campaign 11: 2023‐12‐11 to 2023‐12‐15; campaign 12: 2024‐01‐15 to 2024‐01‐20; campaign 13: 2024‐02‐19 to 2024‐02‐24; campaign 14: 2024‐03‐11 to 2024‐03‐16)	URRUp	1	1	0	0	2
	IRR1	7	0	1	0	8
	IRR2	1	0	0	1	2
	IRR3	0	0	0	0	0
	URRDown	0	0	0	0	0
Total of registers (season 4)		9	1	1	1	12
5 (campaign 15: 2025‐01‐13 to 2025‐02‐25; campaign 16: 2025‐02‐26 to 2025‐03‐18)	URRUp	2	5	2	0	9
	IRR1	4	7	4	0	15
	IRR2	8	1	4	0	13
	IRR3	2	0	2	0	4
	URRDown	0	0	0	0	0
Total of registers (season 5)		16	13	12	0	41

## Appendix 3

*Lontra longicaudis* signs along the Paraopeba river. (A) Otter holt among tree roots; (B) Otter spraint on a fallen tree trunk in the water; (C) Otter track; (D) Otter sighting. Photos: RLM (A–C) and Larissa Lacerda Moraes (D).
